# The Queuine Micronutrient: Charting a Course from Microbe to Man

**DOI:** 10.3390/nu7042897

**Published:** 2015-04-15

**Authors:** Claire Fergus, Dominic Barnes, Mashael A. Alqasem, Vincent P. Kelly

**Affiliations:** School of Biochemistry & Immunology, Trinity Biomedical Sciences Institute, Trinity College Dublin, 152-160 Pearse Street, Dublin 2, Ireland; E-Mails: cfergus@tcd.ie (C.F.); barnesd1@tcd.ie (D.B.); alqasemm@tcd.ie (M.A.A.)

**Keywords:** micronutrient, gut flora, queuine, queuosine, transfer RNA, translation, tyrosine, tetrahydrobiopterin

## Abstract

Micronutrients from the diet and gut microbiota are essential to human health and wellbeing. Arguably, among the most intriguing and enigmatic of these micronutrients is queuine, an elaborate 7-deazaguanine derivative made exclusively by eubacteria and salvaged by animal, plant and fungal species. In eubacteria and eukaryotes, queuine is found as the sugar nucleotide queuosine within the anticodon loop of transfer RNA isoacceptors for the amino acids tyrosine, asparagine, aspartic acid and histidine. The physiological requirement for the ancient queuine molecule and queuosine modified transfer RNA has been the subject of varied scientific interrogations for over four decades, establishing relationships to development, proliferation, metabolism, cancer, and tyrosine biosynthesis in eukaryotes and to invasion and proliferation in pathogenic bacteria, in addition to ribosomal frameshifting in viruses. These varied effects may be rationalized by an important, if ill-defined, contribution to protein translation or may manifest from other presently unidentified mechanisms. This article will examine the current understanding of queuine uptake, tRNA incorporation and salvage by eukaryotic organisms and consider some of the physiological consequence arising from deficiency in this elusive and lesser-recognized micronutrient.

## 1. Queuosine and Its Derivatives

A striking feature of transfer RNA (tRNA) from all organisms is the variety of post-translational modifications that decorate the mature tRNA molecule. These changes can be found at multiple positions on the purine and pyrimidine bases and the 2’-hydroxyl group of the ribose sugar [1]. Currently, 105 different tRNA modifications are described in the RNA modification database, 99 of which are found in eukaryotes [2]. The modifications bring a wealth of structural and functional diversity to the tRNA molecule and range from simple methylation to more dramatic base changes, such as the isopentenylation of adenosine, or the formation of wybutosine and queuosine. Modifications positioned outside the anticodon loop are generally thought to maintain structural integrity and to act as identity determinants for tRNA interacting proteins, whereas those within or proximal to the anticodon loop contribute to the fidelity and efficiency of protein synthesis [3,4].

**Figure 1:**
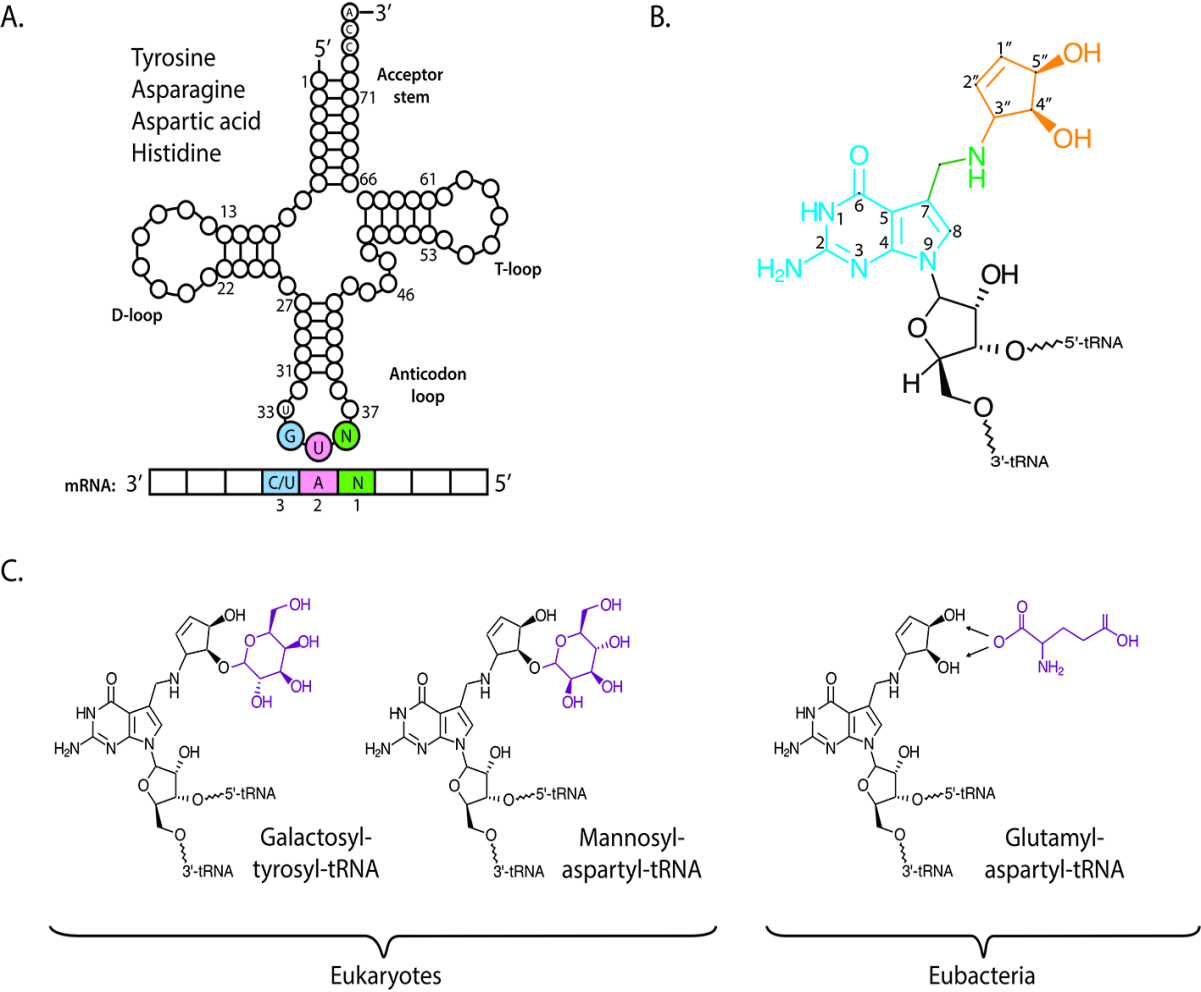
Chemical structure of queuosine and derivatives. (**A**) The G_34_U_35_N_36_ anticodon sequence of tRNA isoacceptors for amino acids tyrosine, asparagine, aspartic acid and histidine will base pair with a N_1_A_2_C/U_3_ codon of messenger RNA (mRNA). G = guanine, U = uridine, A = adenine, N = any base; (**B**) The International Union of Pure and Applied Chemistry (IUPAC) designation for queuosine: 7-(3,4-*trans*-4,5-*cis*-dihydroxy-1-cyclopenten-3-ylaminomethyl)-7-deazaguanosine. Classical nucleic acid numbering is shown. The relative stereochemistries of the cyclopentene substituents have been determined as 3,4-trans and 4,5-cis on the basis of NMR comparisons with synthetic models [22]; (**C**) In mammals, the C4″ hydroxyl of the cyclopentanediol ring of queuosine can be modified with galactose in the case of tyrosyl tRNA and mannose in the case of aspartyl tRNA, by yet unknown enzymes. In eubacteria, the C4″ or the C5″ hydroxyl can be modified by the addition of a glutamic acid residue to its non-cognate aspartyl tRNA by a paralog of glutamyl-tRNA synthetase, glutamyl-Q tRNA(Asp) synthetase (YadB).

Queuosine is among the most elaborate of the known RNA modifications. It was first identified in hydroxylate extracts of tyrosyl tRNA from *E. coli* [5,6,7] and was given the single letter abbreviation of Q, from which the now common name of queuosine- or Q-nucleoside derives. Direct tRNA sequencing methods determined that the queuosine modification is uniquely found in the wobble position of eukaryotic and eubacterial tRNA that contain a G_34_U_35_N_36_ anticodon sequence (tRNA_GUN_; where N = any base), and thus specific to tRNA acceptors for the amino acids tyrosine, asparagine, aspartic acid and histidine (Figure 1A) [8,9] and which decode the dual synonymous codons NAU and NAC. In addition to cytosolic tRNA, the Q modification has also been detected in aspartyl tRNA from the mitochondria of rat and opossum liver by means of the ^32^P-postlabelling technique [10,11]. A related molecule to Q, known as archaeosine, is found at position 15 of the dihydrouridine loop (D-loop) of archael tRNA (readers with an interest in this area are directed to relevant publications [12,13,14]).

Structurally, queuosine comprises a 7-deazaguanosine core (Figure 1B), wherein the purine nitrogen at position seven is replaced by a carbon (in blue), and to which an amino-methyl side chain (green) and cyclopentanediol moiety are appended (orange). In mammals, the hydroxyl group at the C4″ position of the cyclopentanediol ring can be further modified by sugar molecules (Figure 1C); galactose in the case of tyrosyl tRNA and mannose in aspartyl tRNA [15,16]. In contrast to eukaryotes, eubacterial species do not produce sugar-modified queuosine. However the cyclopentene hydroxyl groups of aspartyl tRNA can be modified by the addition of a glutamic acid residue at the C4″ or C5″ position [17,18].

**Figure 2:**
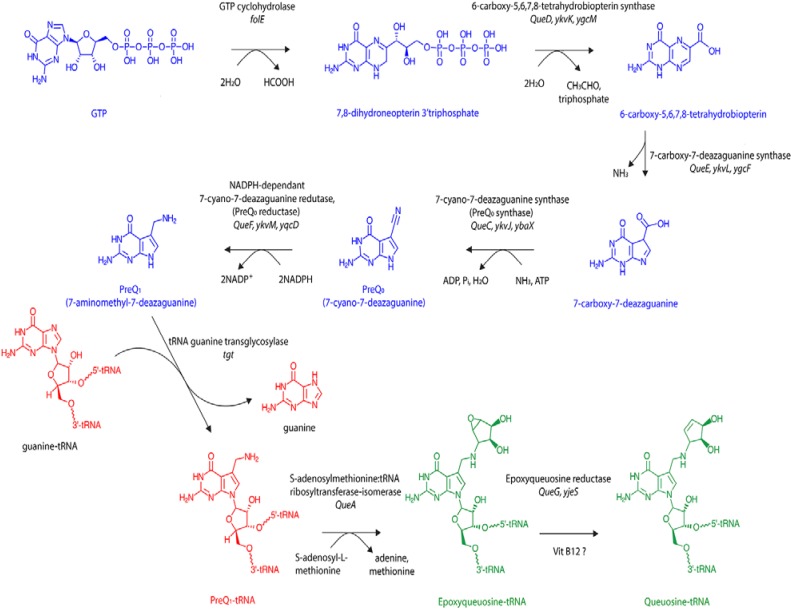
*De novo* biosynthesis of queuosine by eubacteria. Queuosine biosynthesis occurs exclusively in eubacteria *via* initial hydrolysis of the ribose of a guanosine triphosphate nucleoside (GTP) precursor and breakage of the imidazole ring by GTP cyclohydrolase to yield 7,8-dihydroneopterin-3′-triphosphate. In the next two steps triphosphate and acetaldehyde are removed from the pteridine molecule followed by disruption of the pyrazine ring and loss of an amino group to yield 7-carboxy-7-deazaguanine. The fourth and fifth steps in the synthesis are ATP-dependent and NADPH-dependent, respectively, with an aminomethyl group replacing the carboxyl group at position 7 of the 7-deazaguanine molecule to yield the precursor base 7-aminomethyl-7-deazaguanine (PreQ_1_). Eubacterial tRNA guanine transglycosylase then removes guanine from the C1-ribose at the wobble position of the anticodon without cleaving the sugar backbone before inserting PreQ_1_ in a base-for-base exchange reaction. The final two modification steps occur within the context of the tRNA molecule. Firstly, S-adenosylmethionine: tRNA ribosyltransferase-isomerase transfers the ribose moiety from S-adenosylmethionine to the 7-aminomethyl group of PreQ_1_-tRNA. Finally, the oxygen at the C1″ and C2″ of the cyclopentene ring of poxyqueuosine-tRNA is removed in a vitamin B12-dependant reaction to yield queuosine-modified tRNA. Enzyme names are shown in black and below current gene names together with the classical nomenclature from *Z. mobilis* and *E. coli*.

A combination of biochemical and genetic research has, over many years, led to the successful identification of the bacterial enzymes responsible for queuosine biosynthesis [19,20]. The process occurs in two principle phases (Figure 2). Within the cytosol, guanosine triphosphate nucleoside (GTP) is converted to the precursor base 7-aminomethyl-7-deazaguanine (preQ_1_) *via* five enzymatic steps (in blue). This is followed by a transglycosylation reaction (in red) that results in the insertion of preQ_1_ into the wobble position of tRNA_GUN_ isoacceptors concomitant with the displacement of the guanine base. The reaction occurs *via* breakage of the N–C glycosyl bond in a non-energy dependent mechanism that is unique to the tRNA guanine transglycosylase (TGT) enzyme. As such, the transglycosylation reaction represents a signature activity of queuosine formation in all species [19,21]. Two further enzymatic steps function to remodel the preQ_1_ nucleotide *in situ* within the context of the tRNA molecule to give the final queuosine product (in green).

## 2. Natural Sources of Queuosine

The occurrence of queuosine modified tRNA (Q-tRNA) is widespread across the animal and plant kingdoms, yet eukaryotes are unable to synthesize Q-nucleoside or any of its precursor forms. Instead, they salvage the nucleobase of queuosine, referred to as queuine or Q-base. In the case of metazoans the source of queuine is dietary, whether from the gut microflora or from ingested food [9,23,24]. Evidence to support the eubacterial origin of eukaryotic queuosine has been obtained from a number of model organisms. Q-containing tRNA may be fully depleted in *Drosophila melanogaster*, *Caenorhabditis elegans*, *Dictyostelium discoideum* and the eukaryotic algae *Chlorella pyrenoidosa* and *Chlamydomonas reinhardtii* by exclusively maintaining these organisms on a queuine and queuosine-deficient food source [25,26,27,28]. Likewise, mice can be made Q-tRNA deficient by maintaining the animals under germ-free (axenic) conditions and providing a diet lacking any source of queuine or Q-modified tRNA for a period of one year [23].

Attempts have been made to quantify queuine from a number of plant and animal sources (Table 1). These studies have principally relied on the ability of extracts to restore Q-modified tRNA in L-M (mouse fibroblast) cells that have been grown in serum free, and hence queuine-free, conditions [29]. Common foodstuffs, such as yoghurt and milk, contain appreciable amounts of queuine and are likely to be sufficient to meet physiological requirements. In the case of mammalian cells in culture, animal serum proves to be a reliable source, with the exception of horse serum, which due to its low abundance of queuine has been used extensively in research studies to deplete Q-containing tRNA in cells [29,30,31].

**Table 1:** Queuine levels in foodstuff and biological material.

Source	Amount
Yoghurt	4–6 ng g^−1 [32]^
Tomato	21 ng g^−1 [32]^
Coconut water	87–530 ng mL^−1 [32]^
Wheat germ	190 ng g^−1 [32]^
Human milk	1 ng mL^−1 [32]^
Bovine milk (whole and skim)	16–17 ng mL^−1 [33]^
Bovine milk (evaporated skim, canned)	12 ng mL^−1 [33]^
Goat milk (fresh)	3 ng mL^−1 [33]^
Goat milk (evaporated, canned)	1 ng mL^−1 [33]^
Human amniotic fluid	2–84 ng mL^−1 [33]^
Human serum (circulating queuine)	1–10 nanomolar (nM) ^[30]^
Fetal bovine serum	33–54 ng mL^−1 [32]^
Bovine amniotic fluid	2300–3600 ng mL^−1 [32]^
Horse serum	10 nM ^[31]^
Bovine pineal body	300 ng g^−1 [33]^
Bovine seminal vesicle (adult)	110 ng g^−1 [33]^
Bovine testicle (adult)	58 ng g^−1 [33]^
Drosophila melanogaster	0 to 1100 ng g^−1 [33]^

Studies on eubacterial TGT have shown the hydrolysis products of Q-modified tRNA—whether Q-nucleoside, Q-nucleotide or queuine base—are not substrates for the enzyme and therefore presumably, these degradation products will be lost to the gut microenvironment and salvaged by the eukaryotic host as part of the normal turnover process of the microbiome. The proportion of queuine absorbed from the human microbiota has not been determined, but it could be significant given the number of microorganisms in the human gastro-intestinal tract (typically 10^11^–10^12^ microbes mL^−1^ of luminal content; [34]). Furthermore, the relative contribution of the microbiota to queuine supply could be increased in situations where dietary supply is restricted such as during disease and starvation or where a limited variety of food-types are ingested, as in the case of unweaned infants.

Recent efforts have revealed the essential nature of the microbiome to human health and in this context, it is interesting to consider the possibility that the queuosine modification may function to affect the population dynamics of microbial species in the gut. *In silico* studies have predicted that some eubacterial species have lost the genes required for the cytosolic biosynthetic steps (*i.e.*, preQ_0_ and preQ_1_ production) but have retained a functional TGT salvage pathway [19]. Such a scenario is suggestive of a symbiotic or parasitic relationship where biosynthetic intermediates are salvaged from a neighboring species to enhance survival. In this regard, a mutant *E. coli* for the TGT enzyme [35], and an *E. coli* B-strain (B105) naturally deficient in queuosine due to a block in preQ_1_ or preQ_0_ synthesis [36] have both been shown to be rapidly out-competed by queuosine positive strains in mixed culture experiments. Interestingly, pulse-labeling studies with radiolabelled leucine revealed that the mutant Q-deficient TGT strain has a 40% faster rate of protein synthesis than its wild-type isogenic control [35]. Conceivably, due to its positive impact on fitness, the queuosine modification could have a role in quorum sensing and help coordinate biosynthetic processes or population dynamics across the gut microbiome.

## 3. Queuine Uptake and Its Regulation

**Figure 3:**
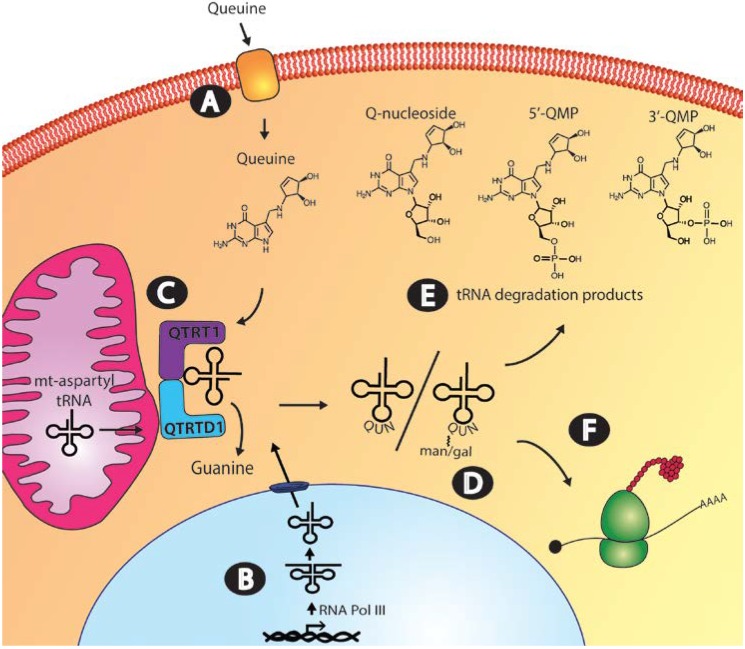
Queuine uptake, incorporation and salvage in the eukaryotic cell. (**A**) Transport of queuine into the cell occurs through a rapidly saturating low *K*_m_ component and a slower-uptake high *K*_m_ component; (**B**) the queuine-insertase enzyme modifies both cytosol and mitochondrial tRNA. Cytosolic tRNA is transcribed in the nucleus by RNA polymerase III, whereas mitochondrial tRNA is transcribed in the mitochondria by mitochondrial-directed RNA polymerase, before processing to the mature tRNA molecule; (**C**) Queuine tRNA-ribosyltransferase 1 (QTRT1) and queuine-tRNA ribosyltransferase domain containing 1 (QTRTD1) subunits of the queuine-insertase complex are cytosolic proteins that appear to co-localize to the mitochondrial membrane where queuine base is incorporated into cytosolic tyrosyl, asparaginyl, aspartyl and histidyl tRNA and mitochondrial aspartyl tRNA. (**D**) Cytosolic aspartyl and tyrosyl Q-modified tRNA can be further modified by the addition of mannose and galactose sugars, respectively. (**E**) Salvage of the queuine base from tRNA turnover could occur from queuosine nucleoside, queuosine-5′-monophosphate (5′-QMP) or queuosine-3′-monophosphate (3′-QMP). Correct functioning of uptake, incorporation and salvage of queuine base is essential to maintaining appropriate cellular Q-modified tRNA levels. (**F**) tRNA_GUN_ recognize the dual synonymous codons ending in either a C or U which pairs to the wobble base.

In humans, it is estimated that normal circulating levels of queuine are in the range of 1–10 nanomolar (nM) [30,37]. Studies conducted on fibroblasts and mammalian cell lines indicate that once in the circulatory system, specific cellular uptake mechanisms operate to move queuine from the extracellular space into the cytosol (Figure 3, Step A) [38,39,40,41,42]. To date, queuine transport has been most extensively studied using human foreskin fibroblasts (HFF) and by exploiting a tritiated derivative of queuine referred to as rQT_3_ [43]. The rQT_3_ derivative displays a similar transporter affinity to its natural counterpart but only 0.1%–0.02% the efficiency for tRNA incorporation ensuring its suitability for such uptake studies [44]. Data from HFF cultures revealed the presence of a biphasic uptake mechanism; a component with a lower *K*_m_ of 30 nM showing rapid saturation within 2–4 min (13.5 pmol (10^6^ cells h)^−1^) and a second transporter component having a higher *K*_m_ of 350 nM and displaying a slower, linear uptake (2.3 pmol (10^6^ cells h)^−1^) that reached equilibrium after 3–4 h [39]. It has been highlighted previously that in comparison to other transport systems for amino acids, nucleosides, and nucleobases, the uptake of queuine is slow [45]. However, the transport mechanism appears to offer significant specificity since various purines, purine-derivatives and base analogues are incapable of affecting queuine transport in competitive uptake experiments [39].

A limited number of reports have indicated that mitogenic signaling pathways may regulate queuine transport. Exposing HFF cultures to chronic levels of the protein kinase C modulator phorbol-12, 13-didecanoate (PDD; 100 nM) was found to decrease Q-modified tRNA [38] concomitant with an increase in the culture saturation density (*i.e*., final density to which a given cell type will grow) to several times that of control [46]. Significantly, the addition of purified queuine (at a concentration of 50 nM) acted to limit the increase in population density caused by chronic PDD exposure and this was concomitant with tRNA being maintained in a queuosine-modified state [38]. The data imply that free queuine base or Q-modified tRNA may be relevant to modulating cell proliferation or culture density [42]. Later studies established that in early passage HFF cultures, PDD and teleocidin (a structurally unrelated stimulator of protein kinase C) were both capable of inducing the inhibition of the high affinity (low *K*_m_) queuine uptake component [45].

Notably, although PDD exposure could induce a large increase in HFF cell number the effect was only transient and not observed in later-passage cultures (5–10 passages; 10–20 population doublings). This was mirrored by an inability of PDD to inhibit rQT_3_ uptake in these later passage cultures [38,39]. Helping to elucidate this difference was the subsequent observation that the media from early passage HFF cultures (but not later cultures) contains a heat-labile, protease-sensitive factor of 10–30 kDa that is responsible for inhibiting queuine uptake [45]. Clues to its identity came from the observation that polyinosinic-polycytidylic acid (poly-IC)—an inducer of interferon synthesis—partially restores the PDD inhibition of queuine uptake in late passage cultures [45]. This led to the demonstration that α-, β-, and γ-interferons are capable of reducing rQT_3_ uptake to a baseline level, approximately 40%–50% that of control cells, with β-interferon proving the most effective [47]. The effects of interferons on uptake were shown to be time and concentration dependent, requiring a pre-incubation period of 24–48 h to elicit a maximal response [47]. Currently, it is not known how extensive the interferon regulation of queuine uptake may be but PDD failed to inhibit rQT_3_ uptake in a number of transformed cell lines including HxGC_3_ (colon carcinoma), CCRF-CEM (T-cell leukemia), HL-60 (promyelocytic leukemia), and Vero (monkey) cells despite the presence of the low *K*_m_ transporter activity [45]. Similarly, unpublished data indicate that conditioned medium from PDD treated early passage HF cells was incapable of inhibiting queuine uptake in L-M, Vero, HeLa, HxGC_3_, CCRF-CEM or HL-60 cell lines [45].

Contrary to the effects seen in early passage fibroblasts, rQT_3_ transport is stimulated by PDD in later-passage fibroblast cultures [45]. This result, and those of subsequent studies, led to a revision of the effects of protein kinase C (PKC) from being an inhibitor to instead being a stimulator for both queuine uptake and incorporation into tRNA. Reconciling these disparate effects is the observation that lower doses and shorter exposure times to PKC activators (determined using 12-*O*-tetradecanoylphorbol-13-acetate; TPA) leads to increased rQT_3_ uptake, whereas chronic long-term exposure has the effect of inhibiting uptake [42]. Likewise, the protein phosphatase inhibitors, okadaic acid and calyculin A, both stimulate rQT_3_ uptake but a cautionary avoidance of excess concentrations and exposure times is necessary [42]. These later studies additionally demonstrated the capability of a wide range of PKC activators to enhance rQT_3_ uptake into primary fibroblasts, including diolein, dicapryyloyl glycerol, phosphatidylserine, and calcium ionophore A23187 [41]. Conversely, inhibitors of PKC activity demonstrated concentration dependent inhibition of rQT_3_ uptake including H-7 (1-(5-isoquinoline sulfonyl)-2-methylpiperazine dihydrochloride), staurosporine, and sphingosine [41,42]. Apart from the aforementioned non-physiological stimulants, a number of growth factors have been shown to positively affect queuine transport across the cell membrane both alone and in combination with PKC activation. For example, in cultured fibroblasts, the uptake of rQT_3_ was increased by the addition of platelet-derived growth factor (PDGF), epidermal growth factor (EGF), and fibroblast growth factor (FGF) to levels approximately 40% above control and the effects of EGF and transforming growth factor β (TGF-β) were found to be additive in combination with PDD [41]. It is important to highlight that, although a major portion of queuine transport appears to be subject to regulation by PKC and mitogenic stimulation, there exists a rather substantial basal rate of uptake, which operates independent of exogenous signaling. Further clarification of the queuine uptake mechanism, its specificity, distribution and regulation awaits identification and characterization of the transporter components.

## 4. Queuine Incorporation into Transfer RNA

Efforts to evaluate the Q-content of tRNA in mammalian tissue have taken advantage of the irreversible nature of the queuosine *N*-glycosidic bond to the tRNA sugar-ribose backbone. The discrimination of G_34_- and Q_34_-containing tRNA_GUN_ is made by the exclusive incorporation of ^3^H-guanine by the *E. coli* enzyme into G_34_-tRNA_GUN_ [48]. By this means, the tRNA of normal adult tissues was found to be highly Q-modified (low ^3^H-guanine incorporation). By contrast, rat pups, fetal liver, and regenerating rat liver were found to contain significant amounts of G-containing tRNA [48,49], hinting at a role for Q-modification in growth and differentiation. A quantitative approach to evaluating Q-tRNA levels provided similar data. Analysis of liver samples form adult rat, beef, sheep and rabbit *via* reverse-phase high performance liquid chromatography all revealed consistently high levels of queuosine (103–127 picomoles of nucleoside per 255 nm absorbance unit; pmol AU^−1^), mannosyl-queuosine (79–104 pmol AU^−1^), and galactosyl-queuosine (30–45 pmol AU^−1^) across adult species [50]. In agreement with earlier reports, the amounts of Q-derivatives in the tRNA of newborn rat liver (1–10 days postpartum) were reduced relative to adult rat: 55% for Q-tRNA, 77% for mannosyl-Q-tRNA, and 46% for galactosyl-Q-tRNA [50]. The fact that newborn and young animals are under-modified with respect to queuosine may be due to the extremely low diversity and number of bacteria present *in utero*, both within the amniotic fluid and the gut of these animals [51], in addition to the highly active cell proliferation and cell differentiation typified by fetal development [50]. Countering this conjecture is the observation that bovine amniotic fluid contains high levels of queuine (2300–3600 ng mL^−1^) and detectable amounts of queuine base have been found in amniotic fluid from normal human pregnancies (16 to 28 weeks gestation) ranging in concentration from 2 to 84 ng mL^−1^ [32]. Given these observations, it is possible that the queuine transglycosylase activity is absent or poorly expressed in early development or that mechanisms exist to limit queuine uptake or its tRNA incorporation *in utero*.

The original attempts to identify the eukaryotic tRNA transglycosylase enzyme relied upon purification from a number of plant and animal sources. The eukaryotic activity is distinguishable from its eubacterial counterpart by the ability to incorporate queuine into tRNA, and is subsequently referred to here as the queuine-insertase enzyme; a term taken from earlier literature [52]. In contrast to the eubacterial TGT enzyme, which is a single protein species, the catalytically active eukaryotic enzyme was purified as a heterodimeric molecule from rabbit erythrocytes [53], bovine liver [54] and rat liver [55] and as a homodimer from wheat germ [56]. The marked differences in the eukaryotic and bacterial enzymes led to the suggestion they may have arisen independently through convergent evolution [57]. However, subsequent to these studies, a cDNA clone encoding a putative catalytic subunit with significant sequence identity to the eubacterial TGT enzyme was shown to re-constitute queuine-insertase activity in GC_3_/c1 cells [58]; a cell line naturally deficient in Q-containing tRNA [59]. This work suggested that in eukaryotes, one of the queuine-insertase component subunits is a homologue of the eubacterial TGT enzyme.

**Figure 4:**
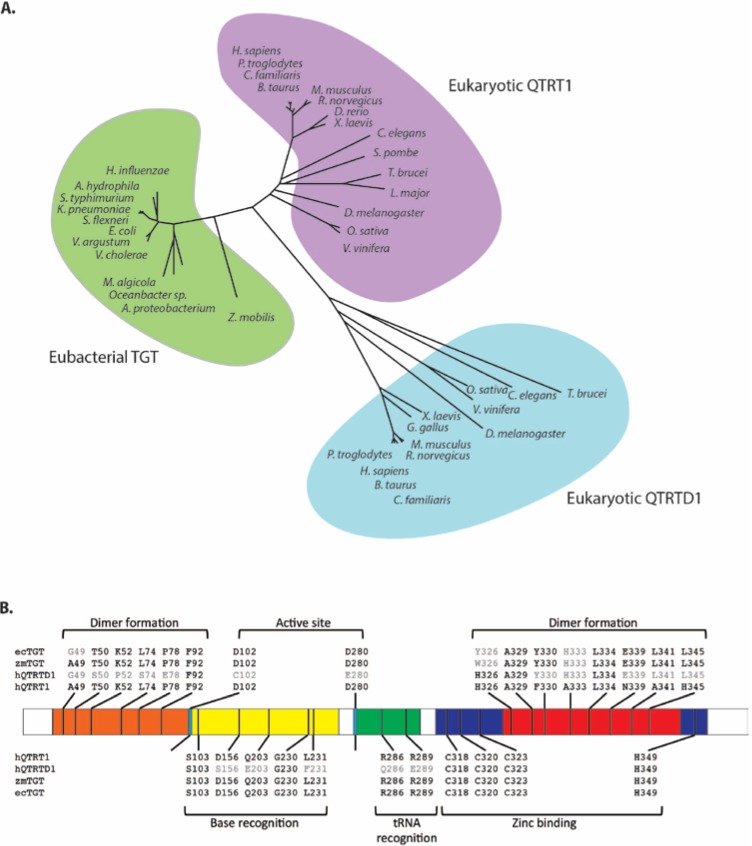
Sequence comparison of Queuine tRNA-ribosyltransferase 1 (QTRT1), queuine-tRNA ribosyltransferase domain containing 1 (QTRTD1) and eubacterial tRNA guanine transglycosylase (TGT). (**A**) Phylogenetic tree showing the relationship of eubacterial TGT (green shading) to eukaryotic QTRT1 (purple shading) and QTRTD1 (blue shading). Unrooted phylogenetic tree generated by comparing the protein sequences of the listed species using Genedoc. (**B**) Significant residues and their corresponding positions within human QTRT1 (hQTRT1), human QTRTD1 (hQTRTD1), *Z. mobilis* TGT (zmTGT) and *E. coli* TGT (ecTGT). Conserved residues are shown in black. The numbering refers to the *Z. mobilis* TGT sequence as described previously [61]. Shown are residues involved in dimer formation in the N-terminus (orange) and the C-terminus (red), residues involved in catalysis: active site residues (light blue), base recognition residues (yellow), tRNA recognition residues (green) and the highly conserved residues involved in zinc binding (purple).

Our own analysis of mouse cDNA and protein databases revealed the existence of two eubacterial TGT related proteins [60]. The first, showing high identity to the human cDNA described above [58], bears the official name queuine tRNA-ribosyltransferase 1 (QTRT1) and is also referred to as the eukaryotic catalytic TGT subunit to denote its equivalence to the eubacterial enzyme. The second protein was more distantly related to eubacterial TGT and is officially annotated as queuine-tRNA ribosyltransferase domain containing 1 (QTRTD1) (Figure 4A). Protein sequence comparisons of QTRT1 and QTRTD1 against eubacterial TGT indicate a probable conservation of secondary structure elements and a strong likelihood they both adopt an irregular (β/α)_8_ triosephosphateisomerase (TIM) barrel structure as previously shown for the *Z. mobilis* TGT enzyme [61,62]. Individually, QTRT1 and QTRTD1 are enzymatically inactive but have comparable guanine transglycosylase activity to the *E. coli* enzyme when mixed in a 1:1 ratio [60]. Studies on the human equivalents of these proteins verified the catalytic requirement for the QTRT1: QTRTD1 heterodimer [63]. Determination of the kinetic constants for tRNA and base revealed a slow turnover rate and *K*_m_ values of 0.34 micromolar (µM) and 0.26 µM for tyrosyl tRNA and queuine, respectively [63,64]. It has been pointed out that the catalytic efficiency of the eukaryotic enzyme is surprisingly sluggish, given the low nanomolar concentrations of queuine normally found in human serum and milk, but this may be offset by the irreversibility of queuine incorporation into tRNA [64].

The QTRT1 catalytic subunit of the eukaryotic enzyme shows a marked conservation of residues with respect to the bacterial TGT enzyme, in particular (numbered according to the *Z. mobilis* TGT) the active site nucleophile Asp280, the general acid/base Asp102, residues linked to tRNA and base recognition and amino acids in the C-terminus involved in zinc-binding (Figure 4B) [60]. Apart from the zinc-coordinating residues, the QTRTD1 subunit differs significantly in sequence from QTRT1 and the bacterial TGT, including a conserved substitution of the active site Asp280 to a glutamic acid. It has been shown in bacteria that the base-for-base exchange reaction occurs through a ping-pong kinetic mechanism and that a covalent intermediate is formed between the tRNA and the Asp280 nucleophile of the enzyme [62,65,66]. Site-direct mutagenesis of the QTRT1 subunit has verified the critical role played by Asp280 in eukaryotes since a QTRT1 mutant renders the queuine-insertase heterodimer devoid of catalytic activity [63]. As explained previously, the eukaryotic enzyme differs from the eubacterial TGT in its ability to utilize queuine as a substrate [21,64].

An explanation for this difference comes from molecular modeling of the *C. elegans* QTRT1 against the *Z. mobilis* TGT structure [67], from kinetic and crystallographic analysis of *Z. mobilis* variants [68] and mutational and kinetic evaluation of the human QTRT1 and *E.coli* TGT enzymes [64]. These studies have determined that in eukaryotes the replacement of Val233 with glycine causes an enlargement of the binging pocket to accommodate the cyclopentanediol moiety of the queuine base whereas the Cys158 to valine change acts to assist in the recognition of cognate substrate (*i.e.*, queuine relative to preQ_1_) [67,68,69].

As no crystallographic data yet exists for the eukaryotic enzyme an explanation of how the individual queuine-insertase subunits function relies on parallels being drawn from eubacterial data. Structural analysis of the *Z. mobilis* TGT has shown dimerization in the unbound and substrate-bound (preQ_1_) state [69,70]. The eukaryotic QTRT1 shows a conservation of dimer forming residues in its N-terminal region and the QTRTD1 subunit shows an almost complete conservation of residues known to form the eubacterial dimer-interphase within its C-terminal zinc-binding domain suggesting a comparable mode of interaction [60]. Further data from *Z. mobilis* TGT using noncovalent mass spectrometry has evidenced the formation of a 2:1 complex with tRNA [71]. It is striking that, despite the presence of two active sites, the *Z. mobilis* dimer can only accommodate one tRNA substrate at a time, arising from steric restrictions imposed by the close packing of the complex [62,71]. Extrapolating from these observations, the QTRTD1 subunit may function as a platform to correctly orientate the tRNA substrate for base-exchange during the QTRT1 mediated catalytic step. It has been suggested that QTRTD1 may also function as a salvage enzyme for queuine by liberating the base from queuosine monophosphate after tRNA turnover [63,72].

An interesting offshoot from the purification studies of the eukaryotic queuine-insertase was the recovery of a number of amino acid sequences with no homology to the catalytically relevant QTRT1 and QTRTD1 subunits. The identity of the proteins from bovine liver [54] were not assigned at publication but our analysis showed that the peptides from the larger 65 kDa subunit are identical to asparaginyl tRNA synthetase and those of the smaller 32 kDa subunit correspond to 2,4-dienoyl CoA reductase. A highly pure preparation from rabbit reticulocytes gave peptides with homology to immunophilin p59, human elongation factor 2 and a deubiquitinating enzyme, USP14 [73]. The identification of USP14 with the larger 65 kDa regulatory subunit has in some cases led to its assignment as a critical queuine-insertase component [74,75] and as being synonymous with QTRT1 by commercial suppliers of antibodies and cDNA clones. Later work ruled out USP14 as a catalytically relevant queuine-insertase component and demonstrated that no physical association occurs with the QTRT1:QTRTD1 subunits [63]. Although the queuine-insertase complex can modify tRNA independent of accessory factors, the co-isolation of the above proteins from purified preparations may indicate that in eukaryotes the transglycosylase activity is embedded in a multi-subunit complex.

Emanating from the PKC studies on queuine uptake, further work revealed that PKC activation might regulate queuine-incorporation into tRNA *in vivo* and be capable of directly phosphorylating and regulating the purified enzyme *in vitro*. Queuine-insertase extracted from rat liver was found to be highly labile under a variety of storage conditions [76]. A suspected requirement for phosphorylation, led to the inclusion of phosphatase inhibitors (sodium fluoride and sodium pyrophosphate) in the isolation buffers, resulting in a dramatic improvement in the recovery of activity during isolation. Likewise, the addition of rat brain PKC together with activatory cofactors (diolein and phosphatidylserine) caused a marked reactivation of queuine-insertase, an effect that was fully reversible by including the PKC inhibitors H-7, staurosporine and sphingosine [76]. That phosphorylation is a major regulator of the enzyme does not reconcile with the fact that active recombinant mouse and human preparations can be isolated from *E. coli* [60,63]. Furthermore, unpublished data from the Garcia laboratory indicates the human queuine-insertase is unaffected by PKC or alkaline phosphatase treatment [63]. Therefore, the phosphorylation effect may relate to associated proteins rather than the core catalytic components. It had been previously proposed that unphosphorylated queuine-insertase exists in a ~104 kDa low-activity dimeric state of two subunits (60 kDa and 34.5 kDa) and that phosphorylation causes the dissociation and release of a highly active catalytic subunit [76]. In these studies, autoradiographic analysis of PKC-treated protein preparations revealed a predominant 60 kDa protein. Conceivably, USP14 or another associated protein could regulate the transglycosylase activity in a PKC-dependent manner.

In cells in culture, both PKC activators (TPA, PDD) and phosphatase inhibitors (okadaic acid, calyculin A) were found to increase the incorporation of rQT_3_ into TCA-precipitated material (tRNA) to levels between 140% and 150% that of untreated control [42]. On the other hand, it was observed that inhibitors of PKC (including sphingosine, staurosporine and H7) decreased incorporation rates of rQT_3_ to levels 40%–50% above baseline [42]. The work suggests that intracellular growth signals are relevant not only to queuine uptake but to tRNA incorporation also. However, it is also apparent from the data that a very significant basal activity of Q-incorporation exists in these cells even in the absence of any further exogenous stimuli.

An unusual effect of PKC-signaling on Q-incorporation has been reported in HeLaS3 cells when grown in horse serum (a serum naturally low in queuine) under low oxygen conditions [77,78]. Cells depleted of queuine by growth in horse serum for 48 h can fully restore Q-modified tRNA if replenished with queuine whether maintained under aerobic or hypoxic conditions. However, if the cells are cultured for an additional 48 h, to deplete serum factors, before replenishment with queuine, hypoxic conditions completely prohibit Q-modification of tRNA, despite the intracellular accumulation of free queuine base. It would appear therefore that a depletion of growth factor signals in combination with hypoxia leads to the inactivation of the queuine-insertase enzyme. The addition of TPA to these hypoxic, queuine-depleted HeLa cultures completely restored Q-modification activity even in the presence of cycloheximide (a protein synthesis inhibitor) and α-amanitin (an RNA polymerase inhibitor) [78]. Similarly, EGF could also restore Q-incorporation and this effect was additive with PDGF, despite PDGF being incapable of restoring modification by itself. The indication that hypoxia negatively affects the Q-modification of tRNA correlates with previous observations that the queuine-insertase enzyme needs oxygen for maximal activity [79]. Whether this effect of hypoxia, like that of phosphorylation, is acting directly or indirectly on the QTRT1:QTRTD1 complex remains to be determined.

## 5. Cytosolic and Mitochondrial tRNA Modification

The original studies describing the eukaryotic queuine-insertase activity date back to 1962: a time when the mechanistic details of translation were still being elucidated. Incubation of intact rabbit reticulocytes with ^14^C labeled guanine led to its incorporation into 4S RNA but not into ribosomal RNA or messenger RNA [80]. Subsequent research by Farkas and colleagues established that the guanine incorporated by rabbit reticulocytes is limited to a specific set of tRNA [81,82], which were later defined by the Nishimura laboratory as the isoacceptors for tyrosine, asparagine, aspartic acid and histidine [83]. In the case of eubacteria, the minimal RNA recognition motif for the TGT enzyme is a 7-base loop containing a U_33_G_34_U_35_ sequence [84], although the specificity determinants for the eubacterial enzyme appear relatively modest. Substrates have been shown to include a non-physiological tRNA dimer [85], the T-arm of an *in vitro*-transcribed yeast phenylalanyl tRNA [86], and a uracil containing DNA stem loop [87]. Other more physiological, but non-tRNA substrates, have also been described for the *E. coli* enzyme. The VacC chromosomal locus of *Shigella flexneri* was shown to be homologous to TGT and mutants lacking this region display a dramatic reduction in virulence due to an attenuation in the translation of the *virF* message [88]. Using the *E. coli* TGT enzyme and a tritiated preQ_1_ substrate, it was shown that the Virulence regulon transcriptional activator (virF) mRNA can be modified *in vitro* at a single base [89]. Other studies exploited tritiated preQ_1_ to show labeling of a number of non-tRNA species in *E. coli*, which was confirmed by *in vitro* reactions, although the identity of these RNA species was not determined [90]. Earlier studies ruled out DNA (from salmon) and ribosomal RNA (bovine and rat liver) from eukaryotic sources as being substrates for the *E. coli* enzyme [48]. With respect to RNA recognition in eukaryotes, *in vivo* microinjection studies of tRNA into Xenopus laevis oocytes showed that, like the eubacterial enzyme, a U_33_G_34_U_35_ sequence in a 7-base loop is essential for enzymatic activity [91]. However, unlike eubacterial enzymes it also appears that eukaryotes require an intact tRNA molecule for efficient transglycosylation [92]. There have been no reports of RNA substrates for the queuine-insertase enzyme beyond the canonical cytosolic and mitochondrial tRNA_GUN_ isotypes.

In eukaryotes, cytosolic tRNA is made by RNA polymerase III as a primary transcript requiring a number of post-transcriptional processing events, including the removal of the 5′ leader and 3′ trailer sequences, splicing to remove introns, the inclusion of the 3′ terminal CCA nucleotides and the addition of modifications (Figure 3, Step B). Enzymes involved in modifying tRNA may be exclusively located in the nucleus, cytoplasm or mitochondria but examples exist where isoenzymes from the same nuclear encoded gene partition to more than one subcellular compartment [93]. An investigation of queuine-insertase distribution in Cos7 (monkey kidney) cells show that the QTRT1 and QTRTD1 subunits are excluded from the nucleus and confocal studies revealed that both subunits co-localize to the mitochondrial membrane (Figure 3, Step C) [60]. The fact that queuine-insertase is not found in the nucleus agrees with oocyte microinjection studies in *Xenopus laevis* wherein cytoplasmic delivery of yeast aspartyl tRNA results in efficient modification with queuine [91,94] whereas nuclear delivery of tyrosyl tRNA led to modest modification levels (~20%) [95]. Notably, in contrast to the confocal data, subcellular fractionation resulted in the QTRT1 catalytic subunit distributing exclusively to the cytosolic fraction. Presumably therefore, the association of QTRT1 to the mitochondria is non-permanent and occurs through direct or indirect binding to QTRTD1 or other associated proteins.

Mitochondria are now understood to influence many diverse and critical cellular processes and their dysfunction is associated with several diseases in humans [96,97,98,99]. Variation occurs across the eukaryotic kingdom, however in humans and other eutherian mammals, the mitochondrial genome encodes a complete set of 22 tRNA species (mt-tRNA). The mt-tRNA can be distinguished from cytoplasmic tRNA through unique sequence and structural features. They perform an essential function in the translation of thirteen subunits of the electron transport chain all of which are vital to meeting the energy requirements of the cell for growth, differentiation, and development (for review see [100]). Therefore, the fact that mt-aspartyl tRNA is a substrate for Q-modification [10,11] suggests queuine could influence a myriad of physiological processes. How Q modification of mt-aspartyl tRNA occurs is presently not understood. It is feasible that a small amount of the queuine-insertase complex exists within the mitochondrial matrix, which is too low in abundance to be detected by Western blot [60]. Alternatively, Q-modification may require mt-aspartyl tRNA to gain access to the outer cytosolic membrane. In this regard, there have been some reports of mitochondrial-encoded tRNA being exported to the cytoplasm in humans [101] and both *in vitro* transcribed glutamyl tRNA and derivatives of lysyl tRNA from *S. cerevisiae* can be imported into the matrix of isolated human mitochondria [102,103].

As described previously, cytosolic Q-modified aspartyl and tyrosyl tRNA are substrates for mannose and galactose modification at the C4"-position of the cyclopentanediol moiety (Figure 3, Step D) [15,16]. It has proven possible to purify these sugar-containing tRNA species by chromatography on lectin-affinity columns that have specificity for the respective sugar [16,50]. Microinjection studies of chimeric tRNA molecules into the oocytes of *Xenopus laevis* indicate a cytosolic location for the glycosyltransferase enzyme and have revealed the enzyme shows sensitivity to the anticodon nucleotides at positions 36–38 [91]. The enzyme responsible for modifying aspartyl Q-tRNA has been partially purified from the soluble fraction of rat liver and shown to accept GDP-α-mannose in the transferase reaction [104]. It is thought that the mt-aspartyl tRNA is not mannosylated, which may be due to the mannosyl-transferase enzyme or its sugar substrate being excluded from the mitochondrial compartment or could possibly result from the mt-aspartyl tRNA being unsuitable to function as a substrate due to incompatible structural features.

## 6. Queuine Salvage

Given the unique dietary dependence that eukaryotes have for queuosine, it is perhaps not surprising that there exists a salvage mechanism to maintain queuosine above critical levels. Efficient salvage ensures that the Q-modification of tRNA could persist under limited queuine supply and decreases the net requirement from the gut. In mice that have been maintained on a queuine and Q-tRNA deficient diet for four weeks the levels of Q-modified histidyl tRNA and asparaginyl tRNA were only depleted to one-sixth that of normal levels and no decrease was observed in the queuosine content of aspartyl tRNA or tyrosyl tRNA [23,105]. As observed in these studies, a distinct hierarchy for queuine incorporation into tRNA_GUN_ species is apparent, with aspartyl tRNA taking precedence over other Q-tRNA isotypes. For example, when fully Q-deficient mice (germ free and fed a defined diet for one year) were administered queuine at a concentration of 0.75 g per gram body weight, this resulted in Q-modification of aspartyl tRNA to 96% but only a 35% modification of histidyl tRNA [23]. Similar results have been seen in cells in culture. Rat liver epithelial cells grown in horse-serum containing medium (low queuine) show a reduction of mannosyl Q-tRNA to 57% normal levels but a drop in galactose Q-tRNA and Q-tRNA to 16% and 9%, respectively [50]. The Farkas group previously suggested that the sugar modification may account for the stability of Q-modification in the case of aspartyl tRNA [105] and speculated that queuine-insertase may display a higher affinity for aspartyl tRNA relative to the other Q-tRNA isoacceptors [23]. In contrast to the effect of PKC on queuine uptake and incorporation the salvage pathway in eukaryotes does not appear to be affected by phosphorylation [76,106].

As mentioned previously, queuine base is the substrate of the queuine-insertase enzyme and therefore, by necessity, salvage must entail the removal of queuine from a nucleoside or nucleotide intermediate following tRNA turnover in the cell (Figure 3, Step E). The eukaryotic queuosine salvage mechanism has been most extensively studied in Vero (African green monkey kidney) cells [43,107]. Pulse labeling Vero cells with tritiated queuine determined the half-life of queuosine in tRNA to be 52 days, indicating the existence of an efficient salvage mechanism and negligible intracellular catabolism [107]. Exogenously supplied Q-nucleoside can function as a substrate for Q-tRNA formation in intact Vero cells [107]. However, crude cell extracts are unable to convert Q-nucleoside to queuine base, or perform the reverse conversion [43], leading the Katze group to hypothesize the existence of a phosphorylated salvage intermediate. Gündüz and Katze exploited the fact that L-M cells are unable to salvage queuosine from degraded tRNA and instead accumulate a large fraction of 5′-QMP intracellularly, which could be isolated for *in vitro* analysis. Vero cell extracts were found to successfully convert the purified 5′-QMP to queuine base, whereas neither 3′-QMP nor mannosyl 5′-QMP were accepted as substrates for salvage [107].

In contrast to the limited activities seen in the cellular extracts, mechanisms must exist *in vivo* to interconvert the various forms of queuosine since exogenously supplied Q-nucleoside can function as a substrate for Q-tRNA formation in Vero cells [107]. This would require the existence of a kinase to convert the nucleoside to 5′-QMP. Indeed, Gündüz and Katze claim to observe such an activity in both L-M and Vero cell extracts [43]. In addition, Vero cell extracts were shown to have the ability to hydrolyse the L-M cell derived 5′-QMP to Q-nucleoside and sugar-modified Q-nucleoside, the production of which is speculated to rely on a membrane bound 5′-nucleotidase. Furthermore, although mannosyl 5′-QMP is not a direct substrate for salvage activity in Vero cells, Gündüz and Katze argue that the sugar residues (mannose and galactose) must be removable *in vivo* since L-M cells contain relatively small amounts of free sugar-modified queuosine (5%) in comparison to the proportion found in tRNA (approx. 50%) [24,43].

Salvage was also investigated in partially purified extracts from the eukaryotic algae, *Chlorella pyrenoidosa* and *Chlamydomonas reinhardtii* yielding somewhat different results [26]. Candidate substrates were added to cell free extracts and products resolved by HPLC. In this case it was observed that queuine was derived from queuosine nucleoside and not 5′-QMP revealing an alternative salvage mechanism in these algae. A search of genes that co-distribute with eukaryotic QTRT1 and QTRTD1 recently identified a potential Q salvage protein, DUF2419 [108]. Evidence for the importance of this protein is provided by the fact Q-modified tRNA is absent from *Schizosaccharomyces pombe* carrying a deletion in the DUF2419 gene and maintained on a bactopeptone containing medium; a known source of queuosine intermediates [26]. DUF2419 is functionally conserved across diverse species and homologues from *Zea mays*, human and *Sphaerobacter thermophiles* were found to complement the gene defect in mutant yeast. Structural modeling determined that DUF2419 bears homology to a DNA glycosylase from the hyperthermophilic bacterium *Pyrobaculum aerophilum* and this together with the spatial location of two invariant basic residues (suitably positioned to interact with a phosphate group) are suggestive that the protein may be a ribonucleoside hydrolase that interacts with 3′-QMP as a substrate or product [108]. Together, the results of these studies indicate that Q-salvage mechanisms may differ across species or alternatively that experimental design has biased the identification of one principle salvage mechanism over another.

## 7. Queuine and Queuosine in Metabolism, Development and Aging

As mentioned above, the studies of Farkas in germfree mice definitively shows that vertebrate species are nonautotrophic for queuosine biosynthesis and under laboratory conditions the animals have no overt pathologies [23,105,109]. However, withdrawal of tyrosine from the diet resulted in dramatic symptoms in these animals that included squinting, stiffness, lethargy, convulsion and invariably death after only 18 days [110]. The re-administration of chemically synthesized queuine or tyrosine completely prevented the symptoms. The data indicate that depriving animals of queuine is lethal in combination with tyrosine despite the fact that tyrosine is a nonessential amino acid in higher eukaryotes and can be synthesized from phenylalanine through the activity of the enzyme phenylalanine hydroxylase (PAH) [111]. However, the levels of PAH or its cofactor tetrahydrobiopterin (BH4) were not examined in these germfree animals.

Genetrap knockout mice for the catalytic QTRT1 subunit (*Qtrt1^Gt^*) are deficient in Q-tRNA and, similar to the germfree mice of the Farkas studies, appear healthy and viable. There were no notable reductions in litter size, fecundity, or lifespan [112]. Surprisingly, their maintenance on a tyrosine free chow diet for two months did not lead to any obvious symptoms of ill health. However, both *Qtrt1^Gt^* mice and queuine deprived HepG2 (human hepatoma) cells were found to have a reduced capacity to convert phenylalanine to tyrosine. This effect was not related to a reduction in PAH protein or activity as a consequence of attenuated translation. Rather, *Qtrt1^Gt^* animals showed a significant decrease in plasma BH4 concomitant with an increase in plasma and urine levels of the oxidized biopterin, dihydrobiopterin (BH2); a known competitive inhibitor of PAH. If changes in BH4 were to occur in the germfree, Q-deficient mice it may partially account for the severe neuropathological symptoms displayed by these animals following tyrosine withdrawal since the BH4 cofactor is critically required for the supply of not only tyrosine but also the neurotransmitters dopamine, epinephrine, norepinephrine, serotonin and nitric oxide.

The underlying cause of BH2 accumulation in *Qtrt1^Gt^* mice is uncertain but may relate to general metabolic changes promoted by queuine deficiency [77,79,113,114]. It is interesting that queuine deprived mice and *Qtrt1^Gt^* animals differ in their sensitivity to tyrosine deprivation, highlighting the non-equivalence of the two experimental systems. Indeed, in comparison to germfree mice, which were fed a chemically defined diet, the *Qtrt1^Gt^* animals could be expected to have both normal gut flora and queuine levels, and additional nutrients provided by the chow diet. Dissecting the exact relationship between tyrosine production, tetrahydrobiopterin and queuine will require further in depth evaluation of the respective animal models. Such investigations would not only shed light on the physiological function of queuine and Q-tRNA but may uncover unanticipated lines of communication along the gut-brain axis.

The relationship between pteridines (the chemical class to which BH4 belongs) and queuine has been recognized for some time. Obvious structural similarities exist as both are derived from GTP nucleotide. Earlier literature described extracts from the *Drosophila melanogaster* mutants ‘brown’ and ‘sepia’ that can inhibit the queuine-incorporation activity of L-M cells [115]. The sepia mutants show abnormal accumulation of sepiaterin, biopterin and pterin, and the latter two molecules were found to directly inhibit L-M cell Q-incorporation into tRNA whereas all three pteridines were found to inhibit queuine-insertase from rabbit erythrocytes, with pterin showing the greatest effect (a *K*_i_ of 9 nM with guanine as substrate) [115]. Conformation of this original data was provided by studies using queuine-insertase activity purified from rabbit reticulocytes demonstrating that pterin, sepiapterin, biopterin and tetrahydrobiopterin were all capable of inhibiting guanine incorporation into tRNA [44]. In culture, pterin was found to inhibit the formation of aspartyl Q-tRNA in mouse fibroblasts (*K*_i_ ~1 μM) [44]. More recently the inhibition of human queuine-insertase by biopterin was evaluated, yielding a *K*_i_ of 8.9 μM [116]. Being an order of magnitude above the *K*_m_ value for queuine (260 nM), this data would suggest that biopterin is not a particularly potent inhibitor of the enzyme [116].

Further studies in murine erythroleukemia (MEL) cells have provided evidence for a relationship between queuine and tetrahydrobioptein *in vivo*. In these cells, induction of erythroid differentiation by DMSO leads to a rapid but transient three-fold induction of tetrahydrobiotperin levels (to a maximum of 40–50 μM), which precedes an approximate six-fold increase in unmodified tRNA_GUN_ by 24 h [117]. The rise in unmodified tRNA was only transient and found to return to almost full Q-modification post-differentiation. Interestingly, administration of *N*-acetylserotonin, a recognized inhibitor of tetrahydrobiopterin synthesis, eliminated the transient elevation of tetrahydrobiopterin in MEL cells and significantly decreased and delayed the accumulation of unmodified tRNA_GUN_ and the cellular differentiation process. The authors demonstrate a 50% inhibition of the rabbit erythrocyte queuine-insertase activity at physiological BH4 concentrations, as was observed during MEL differentiation. However, inhibition of queuine-insertase alone is unlikely to account for the marked drop in the Q-modification of tRNA_GUN_, especially given the long half-life of tRNA (in the order of days) and of Q-modified tRNA (at 52 days; [107]). Most likely, a combination of queuine-insertase inhibition by tetrahydrobiopterin and enhanced proliferation or a large induction of nascent tRNA synthesis by RNA polymerase III accounts for the change.

As described previously, data indicate that the tRNA of pre-natal mice [118] and young rats have low levels of Q-modified tRNA that increase with age [48] suggestive of a role for queuosine in development and differentiation. Induced differentiation of leukemic cells in culture leads to increased Q-modification of tRNA_GUN_ species [119,120,121]. Further studies in invertebrate species have examined changes in Q-tRNA during development and ageing. In the slime mold, *Dictyostelium discoideum*, queuosine was found to influence lactate dehydrogenase activity [122] and the generation of fungal-like aggregates possibly through regulation of cyclic AMP levels [122]. In *Drosophila melanogaster* the amount of Q_34_- relative to G_34_-tRNA was found to dramatically change during metamorphosis [123]. Larval stages show a steady decline in tRNA Q-modification, reaching near full depletion before subsequently recovering as they develop into adult flies [123]. Although these changes in Q-tRNA would appear to correlate with defined morphological processes during the fly life cycle it is also possible that the changes simply reflect alterations in the diet and environment as the flies mature [109,124,125]. Countering these observations in *Drosophila melanogaster*, the tRNA of *Musca domestica* (housefly) exclusively contains Q_34_-tRNA in the larvae and adult stage, whereas in *Lucilia sericata* (green bottle fly) a significant proportion of G_34_-tRNA is found in larvae [126]. Thus, a common regulatory function for Q_34_ anticodon modification in fly development could not be concluded from these studies. However, more recent genomic analysis of the drosophilid lineage may provide a rational for the observed differences (see below).

## 8. Queuine and Queuosine in Cancer

Decreased Q-modification of tRNA_GUN_ has been demonstrated for a large number of neoplastic tissues and cancer cell lines, most exploiting the ability of *E. coli* TGT to insert radiolabelled guanine into unmodified tRNA_GUN_ as described earlier [127]. This has been specifically shown for human colon [59], ovarian [128], brain [129] and lung [130] tumors and in leukemia and lymphomas [131]. It should be noted that the Q-hypomodification is not a universal phenomenon of cancer. Some studies have shown fully Q-modified tRNA in patient neoplastic samples [59,131,132]. Nevertheless, where hypomodification does occur, it appears to correlate strongly with the tumor grade [59,128,129,130,131]. Indeed, separate studies on ovarian and lung cancer showed that patients with low Q-tRNA content have poor long-term survival [128,130]. Whether the loss of the Q-modification in tRNA has a driving influence in neoplasia is unknown, but it would appear unlikely, as neither the queuine deficient germfree mice nor *Qtrt1^Gt^* animals, displayed any increase in tumor formation [110,112].

**Figure 5:**
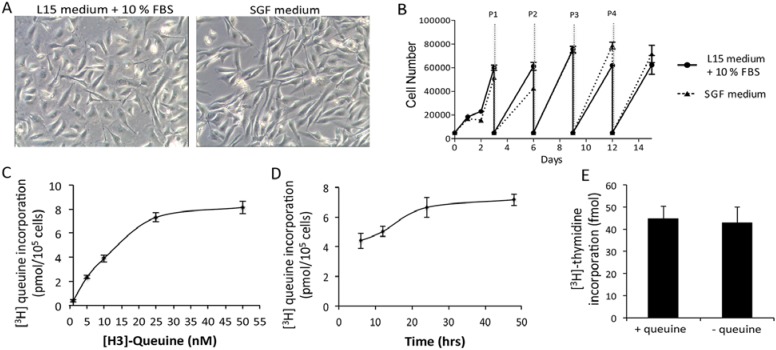

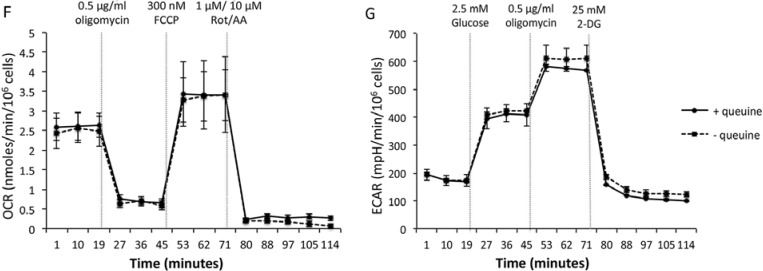
Proliferation and metabolism of queuine deficient human breast adenocarcinoma cell line, MDA-MB-231. (**A**) Growth of MDA-MB-231 cells in Leibovitz L15 medium (10% FBS) is indistinguishable from that in serum-free, growth factor-free medium (SGF medium). (**B**) Cells passaged every three days (P1, P2, *etc*.) on 96-well plates subject to FluoReporter^®^ Blue Quantitation assay. Data are means ± SD (*n* = 8). (**C**) Incorporation of [^3^H]-queuine into MDA-MB-231 tRNA (acid-precipitable fraction) increases with concentration. Data are means ± SD (*n* = 4). (**D**) Incorporation of [^3^H]-queuine into MDA-MB-231 tRNA (acid-precipitable fraction) increases with time. Data are means ± SD (*n* = 4). (**E**) Incorporation of [^3^H] thymidine (0.4 μCi mL^−1^) into MDA-MB-231 DNA is unaffected by treatment with 300 nM queuine over 72 h. (**F**) Oxygen consumption rate (OCR) of MDA-MB-231 cells in response to metabolic modulators is unaffected by treatment with 300 nM queuine over 72 h. Metabolic modulators are: injection 1: 0.5 μg mL^−1^ oligomycin (ATP synthase inhibitor), injection 2:300 nM Carbonyl cyanide-4-(trifluoromethoxy) phenylhydrazone (FCCP) (electron transport chain uncoupling agent), injection 3:1 μM rotenone (electron transport chain complex I inhibitor) and 10 μM (electron transport chain complex III inhibitor). Data are means ± SD (*n* = 4). (**G**) Extracellular acidification rate (ECAR) of MDA-MB-231 cells in response to metabolic modulators is unaffected by treatment with 300 nM queuine over 72 h. Metabolic modulators are: injection 1:2.5 mM glucose (first substrate of glycolysis), injection 2: 0.5 μg mL^−1^ oligomycin (ATP synthase inhibitor), injection 3:25 mM 2-deoxy-d-glucose (inhibits glycolysis). Data are means ± SD (*n* = 4).

The capabilities, if any, that queuosine hypomodification may bestow to a cancer cell, are still poorly understood, and conflicting data exist. In mammalian cells, queuine treatment is reported to modulate tolerance to hypoxia [77], influence proliferation [133,134] and the expression of lactate dehydrogenase [113]. There is a question as to whether the results seen in most of these studies are due to free queuine base or Q-modified tRNA. In HeLa cells cultured in medium containing 10% horse serum, queuine treatment increased cell density under aerobic conditions but decreased cell density under hypoxic conditions [135]. From this work it is suggested that queuine is a stimulant for proliferation in an aerobic environment, but inhibitory when conditions are hypoxic. Later, a study from the same group on the proliferation of non-transformed, transformed and tumor-derived cell lines concluded that queuine can stimulate or inhibit growth, depending on the cell line investigated [134]. In HeLa cells, Kersten’s group could show that under aerobic conditions in the presence of queuine, mitochondrial electron flow was enhanced 1.4-fold, as determined by 3-(4,5-Dimethylthiazol-2-YI)-2,5-diphenyltetrazolium bromide (MTT) assay [77], indicating queuine or Q-modified tRNA may enhance oxidative phosphorylation. Our own unpublished observations failed to see any effect of queuine addition on the proliferation of HeLa, HeLaS3, HepG2, and H4IIE cells grown in commercial serum-free mediums. Furthermore, detailed analysis of the human breast adenocarcinoma cell line MDA-MB-231 grown in a chemically-defined, serum-free medium failed to show any effect of queuine on proliferation (shown by tritiated thymidine incorporation) or metabolism (determined by oxygen consumption and extracellular acidification of the medium) despite being incorporated into tRNA (Figure 5).

The proto-oncogenes *c-myc* and *c-fos* are well established as drivers of increased cell proliferation in neoplastic tissues. The Kersten group observed that in HeLa cells, treatment with queuine caused levels of *c-fos* mRNA to be reduced while *c-myc* mRNA levels were elevated [133]. The group argue that the reduced levels of *c-fos* mRNA are due to increased translation of the protein and therefore destabilization of the mRNA. This study also reports increased levels of protein phosphorylation upon queuine treatment [133]. Friend murine erythroleukemia 745A and M18 cells and human K562 erythroleukemia cells have also been shown to be deficient in queuosine-modified tRNA when in an undifferentiated state [117,119,120,121]. Interestingly, forced differentiation of K562 cells by araC, sodium butyrate, hemin, and azaC led to a restoration in Q-modification of their tRNA [121]. However, differentiation of murine erythroleukemic cells into mature erythroid cells was not induced by the addition of queuine to the tissue culture medium (M. Terade and S. Nishimura, unpublished results; [9]), suggesting that the Q-modification is a result of differentiation, not a causative factor. Q-hypomodification of tRNA could also be induced by transfection of the C3H10T1/2 murine fibroblasts with the Ras oncogene [136].

A number of possible causes for Q-tRNA deficiency in cancer have been proposed including an increased demand for queuine base due to hyperproliferation and rapid tRNA turnover, decreased uptake of queuine due to inhibition of transporters or low bioavailability, defective or absent queuine-insertase activity possibly due to inhibitory biomolecules produced in cancerous cells and tissues, or decreased salvage activity [106]. Limited bioavailability of queuine in rapidly dividing reticulocytes has previously been shown to deplete the levels of queuosine-containing tRNA [137]. Similarly, tRNA in regenerating adult rat liver is hypomodified with queuosine when compared to normal adult rat liver [127,138]. On the other hand, low bioavailability does not provide a full explanation for the loss of the modification, especially in the case of circulatory cancers, leukemia and lymphomas, which would be exposed to normal blood serum levels of queuine. An increased turnover of tRNA in cancerous cells [139], coupled to increased proliferation, could be responsible for the Q-hypomodification in some cancers. In the HxGC_3_ colon adenocarcinoma-derived cell line a complete deficiency of Q-modification was observed compared to HFF cultures and this was attributed to a defective queuine-insertase activity [59]. Treatment of these cells with 5-azacytidine, a DNA methyltransferase inhibitor, increased the incorporation of rQT_3_ into tRNA (0.42 pmol (10^5^ cells)^−1^), albeit at levels five-fold less than that of HFF cultures (2.4 pmol (10^5^ cells)^−1^) [106], leading the authors to conclude that HxGC_3_ cells have a transcriptional defect in the queuine-incorporating enzyme. In MCF7 breast adenocarcinoma cells, which display a Q-deficiency of 50%–60%, the lack of salvage capabilities following turnover of Q-modified tRNA was said to account for the effect [106]. The loss of Q-modified tRNA may also be due to the production of substances in cancerous cells that interfere with the queuine modification system. Pteridine metabolism is altered in neoplastic tissue [140] leading to increased levels of biopterin, neopterin and pterin. Another possible inhibitor of the queuine-insertase enzyme, 7-methylguanine has also been reported to increase in cancer due to higher tRNA turnover [10].

The possibility that the queuosine hypomodification of cancerous tissue relates to enhanced propagation or survival has been addressed by cancer models in animals and cells in culture. In mice administered Ehrlich ascites, the tRNA from tumor-bearing liver samples was found to be Q-deficient and administration of queuine base from bovine amniotic fluid restored the modification in tRNA resulting in an apparent decrease in tumor mass [141]. In mice transplanted with Dalton’s lymphoma ascites cells, administration of synthetic queuine base rescued the Q-modification of cancerous liver tRNA [142] and upon removal of ascites cells from mice, the cell viability seemed to be reduced, as determined by trypan blue staining and cell counting [143]. In contrast to these studies, large amounts of synthetic queuine given by intraperitoneal injection to mice harboring L1210 or S-180 tumors gave no inhibition of tumor growth despite the tumor tRNA_GUN_ being completely restored to Q-containing species ( [9]; unpublished results). It seems unlikely that Q-deficient tRNA is necessary for the neoplastic state. Assessment of the tRNA_GUN_ by RPC-5 chromatography from several Balb/c mouse plasmacytomas showed large variability in the levels of Q-modification even though the plasmacytomas had similar growth rates and size at harvest [144]. The large differences among such a similar group of tumors would argue that the alteration in Q levels is secondary to the neoplastic process.

## 9. Queuosine in Translation

Potentially, Q-modification of tRNA could influence translation at a number of stages (Figure 3, Step F). Studies indicate that the presence of queuosine can affect the efficiency of tRNA aminoacylation. Using rabbit liver aminoacyl tRNA synthetase and unfractionated tRNA preparations from L-M cells it was found that Q-modified aspartyl tRNA had a 30% higher V_max_ and 55% lower *K*_m_ for amino acid charging relative to G-containing aspartyl tRNA [132]. Similarly, the presence of Q_34_ in tyrosyl tRNA from *E.coli* results in a slight decrease in *K*_m_ (0.47 µM) relative to G_34_ (0.6 µM) [35]. The extent to which Q_34_ may influence the rate of amino acid charging across all tRNA_GUN_ species and how this may impact protein translation are presently unknown.

At the level of ribosomal translation, the tRNA_GUN_ isoacceptors are specific for dual redundant codons that vary solely by having either U or C in the third position (*i.e.*, NAC and NAU codons, where N is any base). Therefore, it has long been assumed that the wobble Q_34_ can exert some influence on codon-anticodon recognition. The crystal structure of the 5′-QMP molecule show the cyclopentanediol moiety is positioned such that it would not obstruct Watson-Crick base pairing [145]. Instead, results of *in silico* modeling of aspartyl tRNA suggest Q_34_ acts to restrict the conformational flexibility of the anticodon loop through intramolecular hydrogen bonding [106]. The results of experimental studies in eukaryotes indicate the effects of Q-modification may be subtle. Indeed, no essential difference was observed in the rate or the extent of protein synthesis between Q-containing and Q-lacking tRNA isolated from Drosophila when protein synthesis was carried out in a cell-free, tRNA dependent, mRNA dependent system [125]. Furthermore, Smith and colleagues determined that both Q_34_ and G_34_ histidyl tRNA were capable of distributing labeled histidine equally among all histidine specifying codons in hemoglobin, even in direct competition experiments [146]. Such data indicate that no dramatic differences exist in the ability of G_34_ and Q_34_ modified tRNA to read NAC and NAU codons. The microinjection of histidyl tRNA isoacceptors from Drosophila into *Xenopus* oocytes did, however, yield some notable effects, with G_34_ displaying a clear preference for C- over U-ending codons that contrasts to the results of Q_34_ histidyl tRNA, which exhibits only a slight preference for Q:U pairing over Q:C [147]. A similar conclusion for the effects of G_34_ and Q_34_ were reached in modeling studies of aspartyl tRNA [106].

Redundancy in the genetic code provides scope for the fine-tuning of protein production [148]. Although the impact of Q-modification on codon preference may be modest, across an entire genome the cumulative effect could result in pronounced changes. Data from multiple species have found that the choice of synonymous codons can impact fitness, differing in the speed and accuracy with which they are read as a consequence of tRNA abundance and modification. In this regard, it is interesting that a dramatic shift in codon preference has been identified across the drosophilid lineage and in particular for the amino acids tyrosine, histidine, asparagine and aspartic acid [149,150,151,152]. For example, *D. melanogaster* show a preference for NAC codons whereas *D. virilis* has a preference for NAU codons [153]. This has allowed an interrogation of Q function at the genomic level. Zaborske and colleagues observed that in adult flies, the level of Q-modification provides an accuracy driven selective advantage of C- over U-ending codons. They propose a “kinetic competition model”, wherein the presence of Q_34_ leads to more accurate translation of the C-ending codon as a result of increased binding affinity. This acts to overpower competition from near- and non-cognate tRNA. In the absence of Q_34_, a U-ending codon is more accurate than a C-ending codon since the competition from the wrong tRNA is weaker. In this way, Q-modification acts to reverse the relative codon accuracy within the duel synonymous codon family [153]. If, as they argue, Q-modification is limited by the availability of queuine, one is drawn to the conclusion that this microbial derived micronutrient acts to influence translational fidelity and ultimately the evolutionary fate of the genome of this organism [153].

## 10. Perspective

It is an intriguing thought that queuosine, being so widely distributed across eukaryotic plant and animal species, ultimately (and exclusively) should depend on eubacteria for its production. This perplexing fact, together with queuosine’s central location in anticodon-codon recognition has undoubtedly helped spur enthusiasm for study in this area over many decades. Great advances have been made, especially in recent years where a comprehensive understanding of the queuosine biosynthetic process has been reached, coupled to detailed kinetic and crystallographic analysis of some of the respective enzymes [3,13]. Although falling outside the scope of this review, other fascinating aspects of the eubacterial queuosine system have been uncovered, including the preQ_1_ controlled riboswitches [154,155], and a requirement for the Q biosynthetic pathway for symbiotic colonization of nitrogen-fixing nodules in legumous plants [156].

With regard to the mammalian system, a great deal remains to be understood including the mechanistic processes of queuine acquisition and retention and the cause and consequences of queuosine deficiency under physiological and pathological conditions, such as occurs in early development and cancer. Q modification of tRNA is an ancient process and a compilation of genes essential to the last universal common ancestor have identified the tRNA ribosyltransferase enzyme among the minimal gene set [157]. The results of recent genomic analysis in the *Drosophila* lineage offers tantalizing indications for a role of queuosine in accuracy driven codon preference and in the evolution of the genome of this organism [153]. How such observations translate to other species is an open question. Given its extensive presence throughout the biosphere it is perhaps not surprising that queuosine should leave evolutionary fingerprints that evidence a role in numerous physiological processes. By definition, a micronutrient is a substance required for the normal growth and development of a living organism. Although queuine deficiency in a number of eukaryotic species did not reveal overt ill effects, it is likely that subtle yet essential roles for this elusive micronutrient are only awaiting discovery.
